# Downregulation of miRNA17–92 cluster marks Vγ9Vδ2 T cells from patients with rheumatoid arthritis

**DOI:** 10.1186/s13075-018-1740-7

**Published:** 2018-10-22

**Authors:** Giuliana Guggino, Valentina Orlando, Laura Saieva, Piero Ruscitti, Paola Cipriani, Marco Pio La Manna, Roberto Giacomelli, Riccardo Alessandro, Giovanni Triolo, Francesco Ciccia, Francesco Dieli, Nadia Caccamo

**Affiliations:** 1 Dipartimento Biomedico di Medicina Interna e Specialistica, Sezione di Reumatologia, Università di Palermo, Palermo, Italy; 2 Central Laboratory of Advanced Diagnosis and Biomedical Research (CLADIBIOR), Azienda Ospedaliera Universitaria Policlinico P. Giaccone, Palermo, Italy; 3 Dipartimento di Biopatologia e Biotecnologie Mediche, Università di Palermo, Palermo, Italy; 4 Division of Rheumatology, Department of Biotechnological and Applied Clinical Science, School of Medicine, University of L’Aquila, L’Aquila, Italy

**Keywords:** γδ T cells, Rheumatoid arthritis, Inflammatory cytokines, miRNA17–92

## Abstract

**Background:**

We aimed to evaluate the phenotype, function, and microRNA (miRNA)17–92 cluster expression in Vγ9Vδ2 T-cell subsets and the correlation with immune response in rheumatoid arthritis (RA) patients.

**Methods:**

Peripheral blood from 10 early RA untreated patients and 10 healthy donors (HD) was obtained. Polyclonal Vγ9Vδ2 T-cell lines were generated and analysed by flow cytometry. Analysis of miRNA17–92 cluster expression was performed by real-time polymerase chain reaction (RT-PCR), and expression of mRNA target genes was also studied.

**Results:**

A remarkable change in the distribution of Vγ9Vδ2 T-cell functional subsets was observed in the peripheral blood of RA patients compared with HD, with an expansion of effector subsets and reduction of naive cells which was accompanied by modifications in proinflammatory cytokine expression. Vγ9Vδ2 T cells with a T_EM_ (effector memory) phenotype and producing proinflammatory cytokines were correlated with disease activity score (DAS28). The comparison of miRNA expression among Vγ9Vδ2 T-cell subsets from RA patients and HD showed a lower level of miR-106a-5p and miR-20a-5p, and a higher level of miR-21a-5p, among Vγ9Vδ2 T_EM_ cells, and a lower level of miR-19b-3p among Vγ9Vδ2 T_CM_ (central memory) cells was also found. These differentially expressed miRNAs correlated with higher levels of expression of interleukin (IL)-8, IL-6, and PDCD4 genes.

**Conclusions:**

Our results provide evidence for a role of miR-106a, miR-19-3p, miR-20a, and miR-21a in the regulation of Vγ9Vδ2 T-cell function in RA patients and suggest the possibility that the miRNA17–92 family and Vγ9Vδ2 T cells contribute to the pathogenesis of RA.

## Background

MicroRNAs (miRNAs) are non-coding RNAs (ncRNAs) of around 22 nucleotides in length which play significant roles in regulating gene expression [1, 2]. The miRNA17–92 family is a well-known miRNA cluster involved in health and disease [3] which has long been considered only for its oncogenic role, being in fact known as the first ‘oncomir’ [4]. Several studies have demonstrated a role for this cluster in normal development, immune disease, cardiovascular disease, and many age-related conditions [4]. The cluster is able to maintain a homeostatic setting under physiological conditions essential for the control of inflammatory reactions. Therefore, abnormal expression of this miRNA has been related to several immune disease such as rheumatoid arthritis (RA), and altered miRNA production/expression has been involved in RA pathogenesis [5].

The miRNA17–92 cluster appears as a key factor in the inflammatory pathways activated during RA in synovial cells. It has been widely recognized that an abnormal activation of CD4^+^ lymphocytes producing proinflammatory cytokines (i.e. interleukin (IL)-8, IL-6, IL-17, and tumour necrosis factor (TNF)-α) has a role in RA [6–8], but current studies have also shown that γδ T lymphocytes promote the onset and progression of RA [9]. RA patients show an imbalance between effector subsets (T helper (Th)1, Th17, and γδ T cells) and regulatory T (Treg) cells which likely determines an alteration of homeostasis and favouring a proinflammatory environment [10].

miRNA-mediated RNA interference is emerging as a crucial mechanism in the control of differentiation and function of several lymphocyte subsets, such as γδ T cells, but their specific roles remain to be addressed.

Although the differentiation into various effector subsets and functions of T lymphocytes have been extensively studied, the molecular mechanisms of the differentiation of T-cell subsets and the acquisition of effector function are not completely understood.

We were aimed to evaluate phenotype, effector functions, and miRNA17–92 expression in Vγ9Vδ2 T cells of RA patients compared with healthy donors (HD).

## Methods

### Patients

Heparinized peripheral blood from 10 RA patients (age 40 (range 28–50) years, two female, eight male) and 10 HD (age 43 (27–51) years, three female, seven male) was obtained for this study. Patients fulfilled the 1987 criteria of the American College of Rheumatology (ACR) for RA. All the patients, classified as having an early RA (ERA; disease duration 1.7 years (range 5 months to 2 years), were disease-modifying anti-rheumatic drug (DMARD; methotrexate, leflunomide)-naive and had not received prednisone or equivalent for at least 2 weeks before blood collection. Eight out of the 10 patients were anti-citrullinated protein antibody (ACPA)-positive. Increased levels of erythrocyte sedimentation rate (30.4 ± 15.7 mm/h) and C-reactive protein (0.69 ± 1.2 mg/dl) were also found in all patients. The study was approved by the Ethical Committee of the University Hospital in Palermo where the patients were recruited. Informed consent was signed by all participants.

### γδ T cell identification

Peripheral blood mononuclear cells (PBMC) were obtained by density gradient centrifugation using Ficoll-Hypaque (Pharmacia Biotech, Uppsala, Sweden).

Fc receptor blocking was performed with human immunoglobulin (Sigma; 3 μg/ml final concentration) followed by surface staining with different fluorochrome-conjugated antibodies to study the phenotype and the cytokine production by Vγ9Vδ2 T cells.

The following fluorescein isothiocyanate (FITC)-, phycoerythrin (PE)-, PE-Cy5-, PE-Cy7-, allophycocyanin (APC)-, and APC-Cy7-conjugated anti-human monoclonal antibodies (mAbs) were used to characterise the Vγ9Vδ2 T-cell population: live/dead-FITC, anti-CD45-APCH7 (clone2D1), anti-CD3-PECy7 (clone SK7), anti-TCRVδ2-PE (clone B6), anti-CD27-APC (clone MT271), and anti-CD45RA peridinin chlorophyll protein (PerCP)-Cy5.5 (clone HI100). Expression of surface markers was determined by flow cytometry on a FACSCanto II Flow Cytometer with the use of FlowJo software (BD Biosciences).

For the intracellular cytokine assay, PBMC (10^6^/ml) were stimulated with ionomycin (Sigma, St. Louis, MO, USA; 1 μg/ml final concentration) and phorbolmyristate acetate (PMA; Sigma; 150 ng/ml final concentration). Cells were cultured in a humidified incubator at 37 °C with 5% CO_2_ for 6 h in the presence of 5 μg/ml Brefeldin A (Sigma, St. Louis, MO, USA). Following incubation, PBMC were harvested, washed in phosphate-buffered saline (PBS) containing 1% fetal calf serum (FCS) and 0.1% sodium azide, and then stained as follows: live/dead-FITC, anti-CD45-APCH7 (clone 2D1), anti-CD3-PECy7 (clone SK7), anti-TCRVδ2-PE (clone B6), in incubation buffer (PBS, 1% FCS, 0.1% sodium azide) for 30 min at 4 °C.

Subsequently, PBMC were washed, fixed, and permeabilized (Cytofix/Cytoperm Kit, BD Pharmingen) according to the manufacturer’s instructions and stained for intracellular cytokines with conjugated anti-IFN-γ-APC (clone 25723.11), anti-IL-8-APC (cloneE8N1), and anti-IL-6-APC (clone MQ2-13A5) mAbs. Isotype-matched control mAbs were used. All mAbs were obtained from BD (San Josè, CA, USA) except IL-8 (from Biolegend, San Diego, CA, USA). Cells were washed, fixed in 1% paraformaldehyde, and at least 1 × 10^6^ lymphocytes were acquired using a FACSCanto II Flow Cytometer (BD Biosciences) after gating by forward (FSC) and side scatter (SSC) plots. FACS plots were analysed using FlowJo software (version 6.1.1; Tree Star, Ashland, OR, USA). Negative controls were obtained by staining PBMC in the absence of any stimulation. Cut-off values for a positive response were pre-determined to be in excess of 0.01% responsive cells. Results below this value were considered negative and set to zero [11]. Values found using isotype control mAbs were subtracted in all the samples analysed. The gating strategy used for the phenotype distribution of Vγ9Vδ2 T cells and for the evaluation of the intracellular cytokine content was made starting with the initial lymphocyte gate (SSC vs FSC), followed by gating on single cells, live/dead cells vs CD45, CD3 vs Vγ9Vδ2 T cells, followed by further surface or intracellular molecules.

### Generation of γδ T-cell lines

Polyclonal Vγ9Vδ2 T-cell lines were generated by first enriching PBMC using a γδ T-cell isolation kit (Miltenyi Biotec, Bergisch Gladbach, Germany), followed by sorting single Vγ9Vδ2 T cells through a FACSAria I Cell Sorter (BD Biosciences) with specific mAbs. Sorted cells at the concentration of 2 × 10^4^ were then cultured into each well of round-bottomed plates, containing 2 × 10^4^ irradiated (40 Gy from a caesium source) allogeneic PBMC, plus zoledronic acid (2 μM) and 200 U/ml recombinant IL-2 (Proleukin, Novartis Pharma) [12]. Growing T-cell lines were expanded in 200 U/ml IL-2 and re-stimulated every 3 days. Cells were collected after 2 weeks and sorted according to their phenotype into four different subsets: naive (T_naive_; CD45RA^+^CD27^+^), central memory (T_CM_; CD45RA^−^CD27^+^), effector memory (T_EM_; CD45RA^−^CD27^−^), and terminally differentiated effector memory (T_EMRA_; CD45RA^+^CD27^−^).

### RNA purification and miRNA expression analysis

For analysis of miRNA17–92 among total Vγ9Vδ2 T cells and the different cell subsets, total RNA containing miRNA was purified using an miRNeasy mini-kit (Qiagen). miRNA labelling, hybridization, scanning, and expression profiling was performed using miRCURY LNA microarray service (Exiqon).

### Real-time polymerase chain reaction (RT-PCR) analysis of the whole population of Vγ9Vδ2 T cells

Total RNA was extracted from γδ T-cell lines derived from RA patients and HD with the miRNeasy Mini Kit (Qiagen) isolation kit according to the manufacturer’s instructions. The quality of RNA was accessed with a NanoDrop 1000 Spectrophotometer V3.7 (Thermo Scientific). The obtained RNA was subsequently used as a template for cDNA generation. For this purpose, a reverse transcription reaction was performed with miScript II RT Kit (Qiagen; 300 ng of RNA per reaction) following the manufacturer’s protocol. The resulting cDNA was used to conduct an RT-PCR reaction with miScript SYBR Green PCR Kit (Qiagen) applying primers specific for hsa-miR-21a-5p, hsa-miRNA-hsa-miR-19a-3p, hsa-miR-19b-3p, hsa-miR-20a-5p, and hsa-miR-106a-5p (commercially available from QIAGEN) in a Rotor-Gene Q system. The expression level of RNU6 was used as an endogenous control.

RT-PCR was also performed to evaluate IL-8, IL-6, and programmed cell death 4 (PDCD4) mRNA using the commercially available Illustra RNAspin Mini Isolation Kit (GE Healthcare*,* Little Chalfont*,* Buckinghamshire, UK) according to the manufacturer’s instructions. For quantitative TaqMan RT-PCR, master mix and TaqMan gene expression assays for *GAPDH* (glyceraldehyde 3-phosphate dehydrogenase, Hs99999905_m1) control and target genes were obtained from Applied Biosystems. Samples were run in duplicate using the Step-One Real-Time PCR system (Applied Biosystems, Foster City, CA, USA). Relative changes in gene expression between paired patients before and after treatment were determined using the ΔΔC_t_ method. Levels of the target transcript were normalized to a GAPDH endogenous control constantly expressed in both groups (ΔC_t_). For ΔΔC_t_ values, additional subtractions were performed between untreated and treated samples ΔC_t_ values. Final values were expressed as fold of induction (FOI).

### Statistical analysis

miRNA microarray data were analysed by miRCURY LNA microarray service (Exiqon). Data were normalized using the non-parametric regression method, LOESS. Unsupervised two-way clustering of miRNAs and samples was performed on log_2_ (Hy3/Hy5) ratios (with each sample versus the common reference pool) to produce a heat map. Heat map expression data were displayed using Gene-E software developed by Joshua Gould (http://www.broadinstitute.org/cancer/software/GENE-E). Hierarchical clustering using one minus Pearson’s correlation was applied to samples and genes/miRNAs. Global or relative map colours were applied using the minimum and maximum values in the data. Network analysis to identify miRNA targets using gene and miRNA expression data was performed using MIR@NT@N [13].

Obtained C_t_ values were used to calculate expression levels of tested miRNAs with the 2^ΔΔCt^ method in two groups, each composed of HD and RA patients. To assess the statistical significance of observed differences, independent student *t* tests and Mann Whitney tests were performed on all groups and *p* values *p ≤ 0.05, **p ≤ 0.01, and ***p ≤ 0.001 were considered as significant, very significant, and extremely significant, respectively.

The normal distribution of the data was assessed by a Shapiro-Wilk normality test. Analysis of variance (ANOVA) was performed as part of the data analysis, and these data are reported as a heat map.

## Results

### Skewed distribution of circulating Vγ9Vδ2 T cells in RA patients

**Fig. 1:**
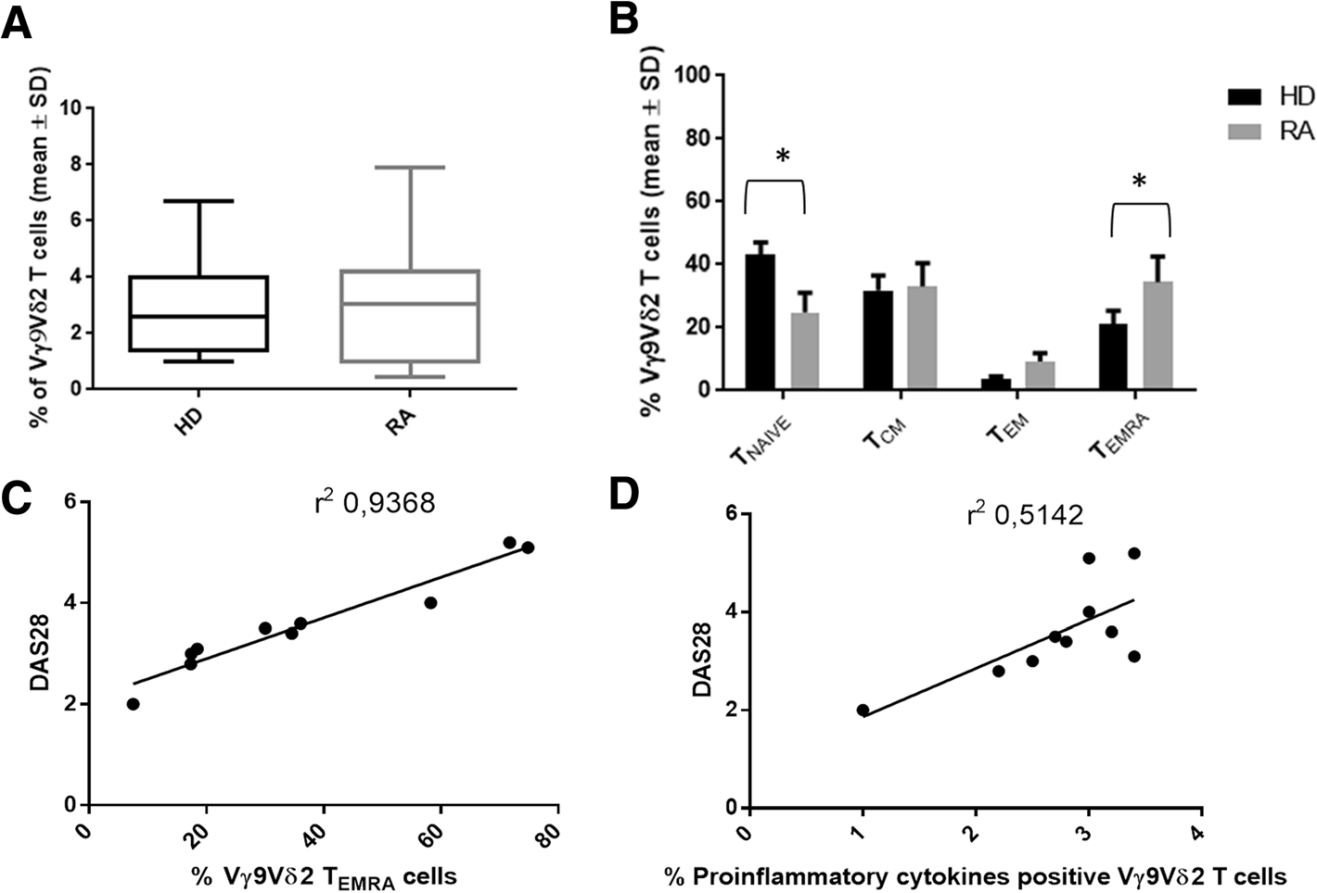
Percentage and phenotype distribution of Vγ9Vδ2 T cells in rheumatoid arthritis (RA) patients and healthy donors (HD) and correlation of T terminally differentiated effector memory (T_EMRA_) cells and cytokines production with activity disease score (DAS) in RA patients. **a** Percentage of Vγ9Vδ2 T cells evaluated in peripheral blood of HD (black box) and RA patients (grey box). **b** Phenotype distribution of Vγ9Vδ2 T cells in HD (black column) and RA patients (grey column). *p < 0.05. **c** Correlation between DAS28 activity scores and the percentage of Vγ9Vδ2 T_EMRA_ cells. **d** Correlation between DAS28 activity scores and the percentage of Vγ9Vδ2 T cells expressing proinflammatory cytokines. T_CM_ T central memory, T_EM_ T effector memory, T_naive_ T naive

Although the mean frequency of peripheral blood Vγ9Vδ2 T cells was similar in RA patients and HD (Fig. 1a), a remarkable change in their phenotype distribution was observed. T_EMRA_ and T_CM_ cells were the major Vγ9Vδ2 T-cell subset in the peripheral blood of RA patients, while T_naive_ and T_CM_ cells were the dominant populations in HD; other Vγ9Vδ2 T-cell subsets were poorly represented in both patients and controls (Fig. 1b). Moreover, we found a statistically significant decrease in T_naive_ and an increase in T_EMRA_ cells when RA patients were compared with HD (Fig. 1b). Most notably, in RA patients the DAS28 activity scores was directly correlated with the percentage of Vγ9Vδ2 T cells with a T_EMRA_ phenotype (Fig. 1c) and expressing the proinflammatory cytokines interferon (IFN)-γ, IL-6, and IL-8 (Fig. 1d).

**Fig. 2:**
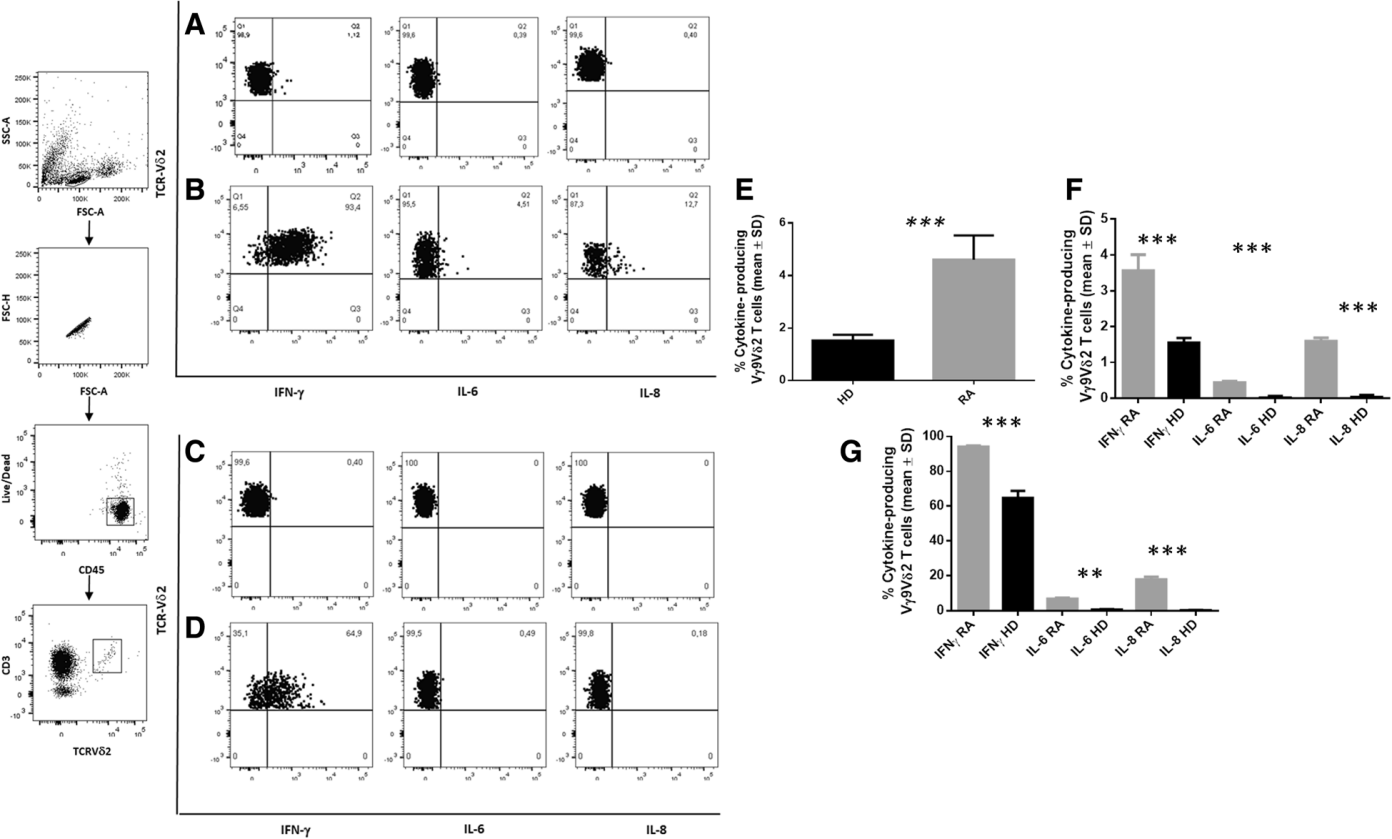
Production of proinflammatory cytokines by Vγ9Vδ2 T cells. Panels on the left show the gating strategy. **a–d** Ex-vivo analysis of cytokine-producing Vγ9Vδ2 T cells. **a** Basal level of cytokines and **b** after ionomycin-PMA stimulation of one representative rheumatoid arthritis (RA) patient; **c** basal level of cytokines and **d** after ionomycin-PMA of one representative healthy donor (HD). **e** Comparison of proinflammatory cytokines (interferon (IFN)-γ, interleukin (IL)-6, and IL-8) production as a total frequency of cytokine-producing Vγ9Vδ2 T cells between HD (black column) and RA patients (grey column). **f,g** Ex-vivo analysis of cytokine-producing Vγ9Vδ2 T cells among RA patients and HD (**f**) and after ionomycin-PMA stimulation (**g**). The mean percentage of Vγ9Vδ2 T cells expressing IFN-γ, IL-6, or IL-8 among 10 RA patients and 10 HD. **p < 0.01, ***p < 0.001. FSC forward scatter, SSC side scatter

We then analysed the ability of Vγ9Vδ2 T cells to produce proinflammatory cytokines, such as IFN-γ, IL-6, and IL-8, by intracellular FACS analysis ex vivo and after short-term in-vitro stimulation with ionomycin and PMA. The left hand panels in Fig. 2 show the gating strategy used to select Vγ9Vδ2 T cells and the sequential gating on lymphocytes, single live cells, live/dead cells/CD45^+^ and CD3^+^ cells vs Vγ9Vδ2 T cells. Figure 2a and b show representative intracellular FACS analysis of Vγ9Vδ2 T cells producing IFN-γ, IL-6, and IL-8 in one representative RA patient ex vivo and after ionomycin and PMA stimulation, while Fig. 2c and d show a representative HD ex vivo and after ionomycin and PMA stimulation.

Figure 2e shows that the percentage of Vγ9Vδ2 T-cell response for the production of total proinflammatory cytokines (IFN-γ, IL-6, and IL-8) in RA patients was significantly elevated compared with HD. Figure 2f and g show the cumulative mean percentage of each cytokine-producing Vγ9Vδ2 T cells in 10 RA patients and 10 HD ex vivo and after stimulation with ionomycin and PMA, respectively.

### Expression of miRNA17–92 in Vγ9Vδ2 T-cell subsets

**Fig. 3:**
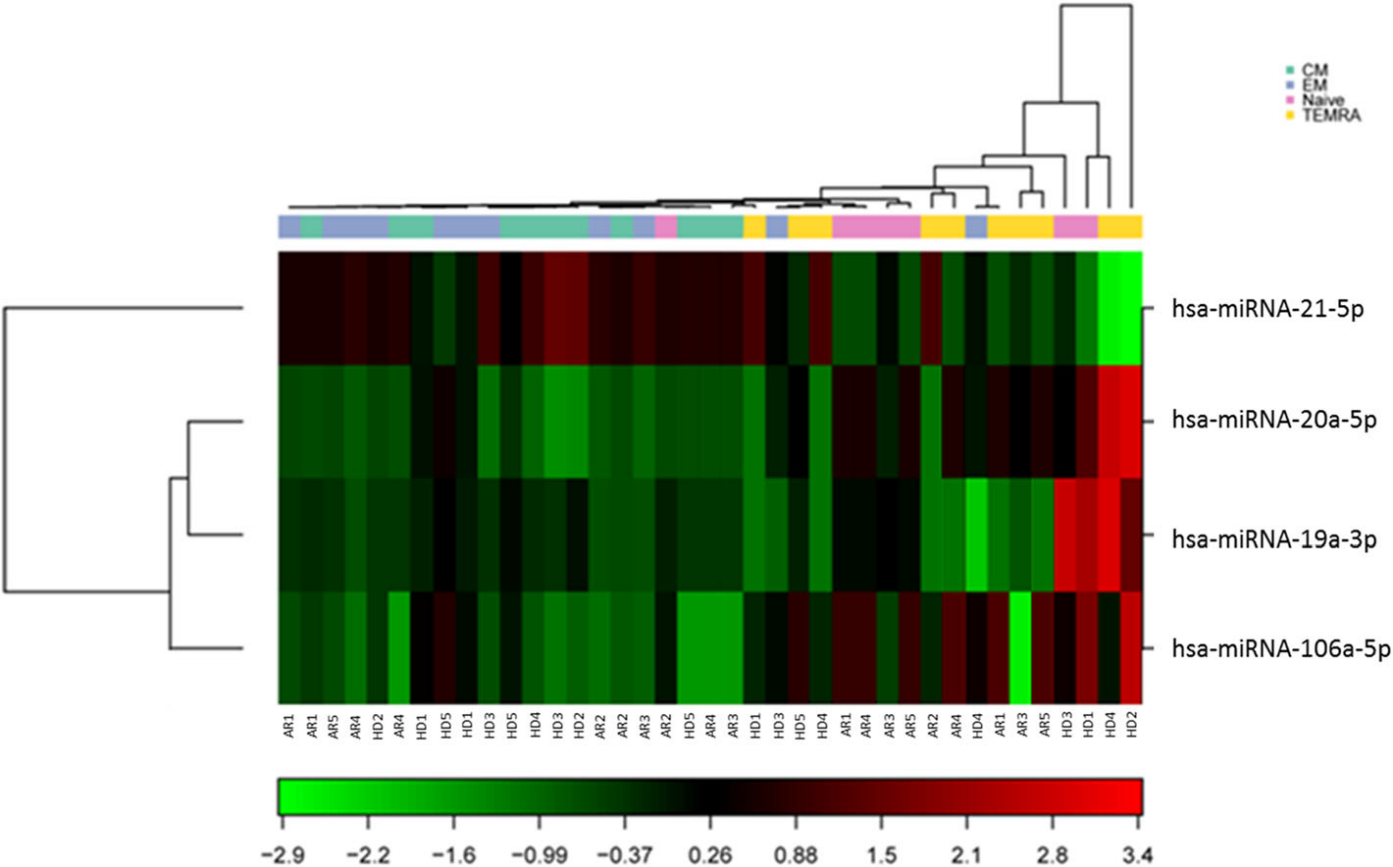
Heat map of the different miRNA expression profiles for each RA patient and HD. The heat map diagram shows the result of the two-way hierarchical clustering of miRNAs in samples from five RA patients and five HD. Each row represents one miRNA, and each column represents one sample. The miRNA clustering tree is shown on the left. The colour scale shown at the bottom illustrates the relative expression level of an miRNA across all samples: red represents an expression level above mean, and green represents an expression lower than the mean. The clustering is performed on all samples, and miRNAs displayed are the large-magnitude changes that are also statistically significant on the five out of 19 miRNAs with the highest standard deviation. Normalized (dCq) values have been used for the analysis. CM central memory, EM effector memory, TEMRA T terminally differentiated effector memory

Vγ9Vδ2 T-cell lines were obtained from RA patients and HD after two weeks of in-vitro culture and were used either as a total population or were further sorted into different naive, memory, and effector subsets for miRNA17–92 expression analysis. Figure 3 shows the heat map of the miRNA expression profile for five RA patients and five HD. When comparing the groups within ‘group’ using a one-way ANOVA, five miRNAs were found to be differentially expressed using a cut-off *p* value < 0.05.

**Fig. 4:**
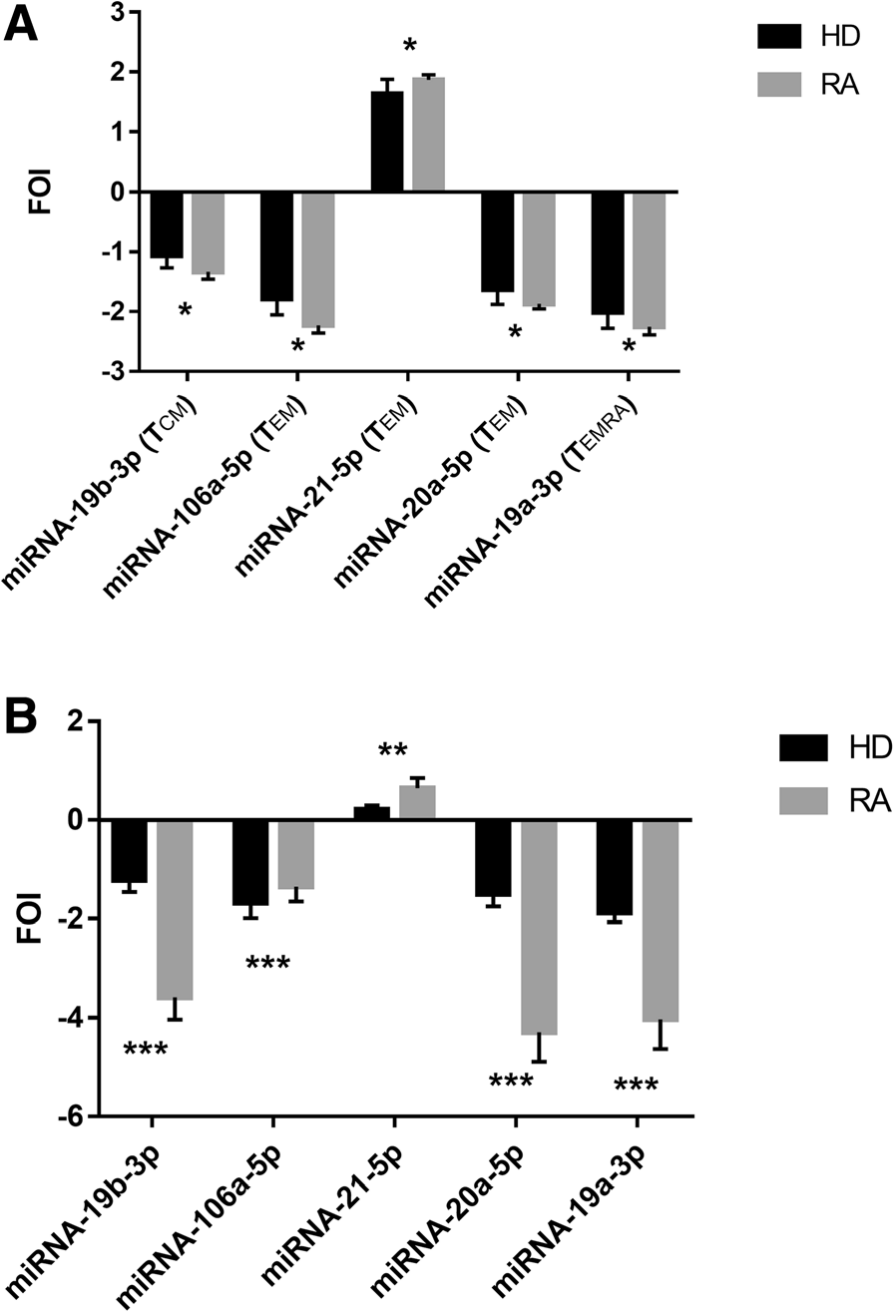
miRNA expression profile in rheumatoid arthritis (RA) patients and healthy donors (HD). **a** Comparison of miRNA expression by different subsets of Vγ9Vδ2 T cells in 10 RA patients and 10 HD. Values indicate fold of increase (FOI) **±** SD. *p < 0.05. **b** Comparison of miRNA expression in the total Vγ9Vδ2 T cells from RA patients and HD. Values indicate FOI **±** SD.*p < 0.05, **p < 0.01, ***p < 0.001. T_CM_ T central memory, T_EM_ T effector memory, T_EMRA_ T terminally differentiated effector memory, T_naive_ T naive

miRNA levels were evaluated as the fold increase or decrease comparing RA patients with HD in the four subsets of Vγ9Vδ2 T cells, where they displayed different expression levels: T_EM_ cells showed lower levels of miR-106a-5p and miR-20a-5p and higher level of miR-21a-5p, while significantly lower levels of miR-19 were found in T_CM_ and T_EMRA_ cells (Fig. 4a). Figure 4a and b show cumulative data from 10 RA patients and 10 HD in the different subsets (Fig. 4a) and in the total Vγ9Vδ2 T-cell population (Fig. 4b), respectively. We did not find any significant differences in miRNA expression in the T_naive_ subset from RA patients and HD. All the other tested miRNAs did not show any significant modulation in all the samples studied (data not shown). The same trend of miRNA expression in selected subsets was also detected when analysing the total Vγ9Vδ2 T-cell population, indicating that the expression of these miRNAs could represent a specific signature of the whole Vγ9Vδ2 T lymphocyte compartment (Fig. 4b).

**Fig. 5:**
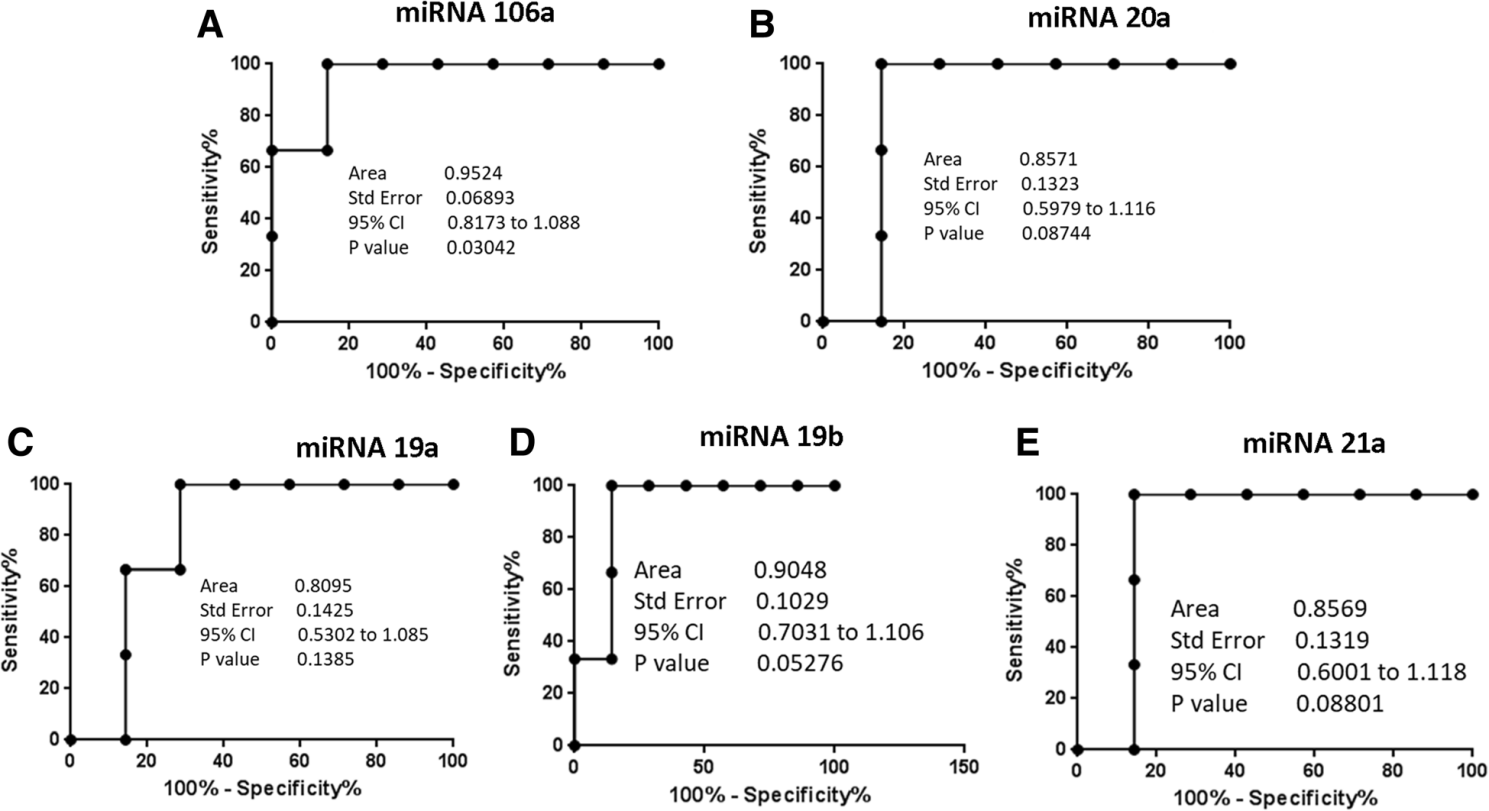
Receiver operating characteristic curves for miR-106a, miR-20a, miR-19a-b, and miR-21a. The solid line shows the results for the value of **a** miR-106a, **b** miR-20a, **c** miR-19a, **d** miR-19b, and **e** miR-21a, comparing RA patients with HD. CO confidence interval

To further assess the accuracy of the miRNA signature of Vγ9Vδ2 T-cell lines as a determinant to discriminate between RA patients and HD, receiver operating characteristic (ROC) curves and cross-over plots were produced. As shown in Fig. 5a, the different miRNAs distinguish RA patients from HD, with the best accuracy for miR-106a (area under the curve (AUC) 0.95, *p* < 0.030, with 91.04% and 93.55% sensitivity and specificity, respectively).

### Correlation of miRNA expression with cytokine and cell survival gene expression

Several miRNAs play a role in the regulation of cytokine gene expression and on the regulation of genes that are involved in cell survival. Therefore, we analysed if the different miRNA profiles found in patients with RA could be correlated with the expression of genes involved in the modulation of the immune response.

**Fig. 6:**
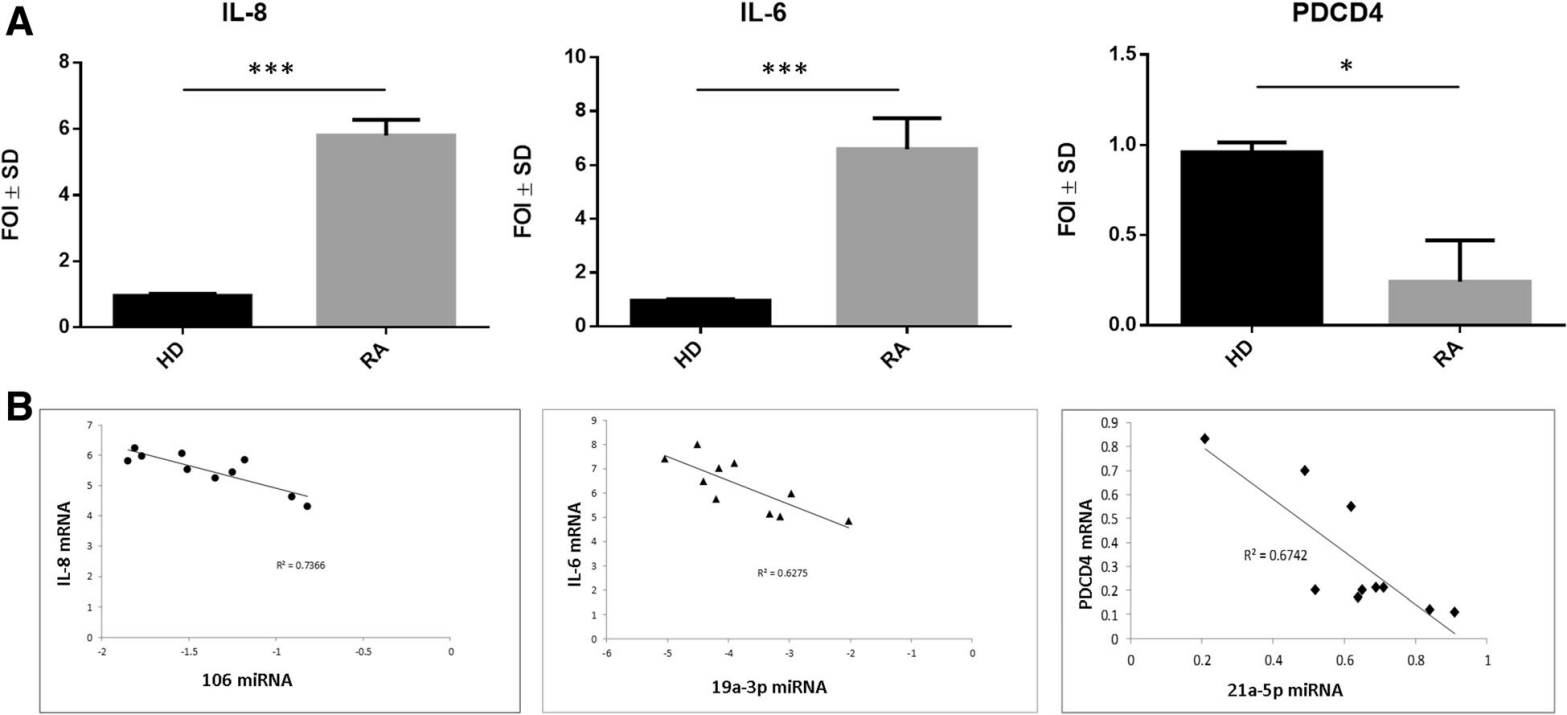
Expression of interleukin IL-6, IL-8, and programmed cell death 4 (PDCD4) mRNA by Vγ9Vδ2 T cells in rheumatoid arthritis (RA) patients and healthy donors (HD). **a** Comparison of IL-6, IL-8, and PDCD4 mRNA expression in Vγ9Vδ2 T cells between HD (black column) and RA patients (grey column). *p < 0.05, **p < 0.01, ***p < 0.001. **b** Correlation between miRNA expression and IL-6, IL-8 and PDCD4 mRNA expression

The comparison of the mRNA levels of the inflammatory cytokines IL-6, IL-8, and PDCD4 gene between RA patients and HD showed statistical significance in terms of fold increase in RA patients (Fig. 6a). Since IL-17 plays a (controversial) role in the pathogenesis of RA [14], we also evaluated IL-17 mRNA levels among γδ T cells, but we found that IL-17 mRNA levels were undetectable in γδ T cells from RA patients and HD (data not shown). Therefore, and considering the important role of miR-106a, miR-19a-3p, and miR-20a-5pin targeting IL-8 and IL-6 genes and the well-known over-expression of IL-6 and IL-8 in RA synovial tissue, we correlated the expression of IL-6, IL-8, and PDCD4 mRNA with these miRNAs and also with miR-19b-3p and miR-21a-5p that were found to be statistically significant in RA patients. Lower levels of miR-106a expression correlated with high levels of IL-8 mRNA in RA patients compared with controls (Fig. 6b), and lower levels of miR-19a-3p correlated with high levels of expression of IL-6 mRNA. An inverse correlation between the expression of miR-21a and the anti-apoptotic gene PDCD4 was also found in RA patients compared with HD (Fig. 6b). We did not find any significant correlation of the above cytokines and PDCD4 gene expression when comparing miR-19b-3p and miR-20a-5p (data not shown). Overall, these data highlight the role of miRNA in patients with RA in the production of inflammatory cytokines, and on the ability of Vγ9Vδ2 T cells to survive and display a potentially pathological role.

## Discussion

The role of miRNA17–92 has been evaluated in different human immune cells such as B, T, and natural killer (NK) lymphocytes, macrophages, and dendritic cells, but its role in γδ T cells is not well understood. The aim of the present study was to investigate the different expression of members of the miRNA17–92 family among Vγ9Vδ2 T-cell subsets from healthy donors and patients with RA [15]. We found lower levels of miR-106a, miR-19a, miR-19b, and miR-20a expression, and a higher level of miR-21a expression in RA patients compared with HD, either in the different subsets or in the total γδ T-cell population.

Vγ9Vδ2 T-cell subsets are characterized by distinct migratory routes and display different functional properties depending on the microenvironment due to their high plasticity [16–18].

The role of Vγ9Vδ2 T cells has been investigated under physiological and pathological conditions such as infections, autoimmunity, or cancer. Therefore, we aimed to evaluate the role of the miRNA17–92 cluster on Vγ9Vδ2 T-cell functions in RA patients to uncover a biosignature of disease. The analysis of the phenotypic distribution and functional properties of γδ T cells showed remarkable changes in RA patients. In fact, T_EMRA_ and T_CM_ cells were the predominant Vγ9Vδ2 T-cell subsets in the peripheral blood of RA patients and these cells represented a relevant source of proinflammatory cytokines.

The comparison between the levels of proinflammatory cytokines and Vγ9Vδ2 T cells with the T_EMRA_ phenotype correlated with the severity of the disease.

Different miRNA17–92 levels were expressed both in the total population and in the different subsets of the Vγ9Vδ2 T-cell population.

Five out of 19 miRNAs displayed a large magnitude of change in RA patients. Therefore, to evaluate if the expression levels of these miRNAs could be considered as biomarkers of disease, we studied the accuracy by ROC curve analysis. The results demonstrated that the best performance was found for miR-106a, followed by miR-20a, miR-19a, miR-19b, and miR-21a.

Therefore, we correlated the IL-8 mRNA expression with miR-106, and IL6 mRNA levels with miR-19a levels to find direct evidence of the modulation of these two proinflammatory cytokines and their role in the contribution to inflammation and joint damage. Accordingly, lower levels of miR-19 in our RA patients correlated with the increase in IL-6, and this was in agreement with a previous study showing that treatment with IL-6 but not with TNF-α led to down-regulation of miRNA19 expression [19]. In-vitro studies have demonstrated that IL-8 is a direct target of miR-106a, and an miR-106a inhibitor increases its production [20]. In the absence of miR-106a, fibroblast-like synoviocytes (FLS) from RA patients produce more IL-6 and IL-8, which contribute to inflammation and joint damage.

We have found that the lower levels of miR-106a, miR-20a, and miR-19 were accompanied by higher levels of miR-21a in RA patients.

We have found an inverse correlation of miR-21a levels with PDCD4 mRNA levels. The elevated levels of miR-21 could induce T-cell proliferation by negatively regulating PDCD4 gene expression [21], as observed in our RA patients, by causing the sustained production of proinflammatory cytokines by cells that upregulate the PDCD4 gene, which is correlated with a high survival rate. Since aberrant expression of miR-21 is related to increased susceptibility to immune-inflammatory disorders, an adequate expression of miR-21 might be critical for regulating normal immune responses. Evidence is now accumulating to support the therapeutic potential of miR-21 in autoimmune disorders.

Finally, the upregulation of miR-21 could be sustained by the contextual downregulation of miR-20a that could contribute to the lack of an anti-inflammatory property [22].

It has been demonstrated that higher levels of miR-21a occurs in all types of solid tumours, and additional studies showed elevated miR-21 expression also in leukaemias [23]. Interestingly, high levels of miR-21 may not only characterise cancer cells but also represent a common feature of cell stress as demonstrated by Xu et al. on the correlation between inflammation and cancer [24].

We do not know if the reduced expression of miR-20a could contribute to the lack of anti-inflammatory responses. miR-20a represses ASK1, a member of the kinase family that is activated in response to several stress signals, including lipopolysaccharide (LPS) or TNF-α. Activated ASK1, in turn, generates the production of reactive oxygen species (ROS) by a NADPH oxidase 4 (Nox4)-dependent mechanism, and consequently by decreasing the capacity of FLS to secrete IL-6 or matrix metalloproteinase (MMP)-3 [25].

Therefore, the decrease or increase in the tested miRNAs could impact at different stages of RA, with the production of proinflammatory cytokines and the maintenance of cells due to their high survival rate because of the negative regulation of PDCD4 altogether contributing to inflammation in RA patients.

It has been demonstrated that overexpression of miR-20a in human naive CD4^+^ T-helper cells inhibits TCR-mediated signalling and CD69 expression, and determines the decrease in production of cytokines such as IL-6 and IL-8 [22]. Therefore, we speculate that miR-20a could also be implicated in the control of proinflammatory cytokine production by γδ T cells.

## Conclusions

Our results provide evidence for a role of miR-106a, miR-19a-b, miR-20a, and miR-21a in the regulation of Vγ9Vδ2 T-cell functions in RA patients and suggest the possibility that the miRNA17–92 family and γδ T cells could be involved and contribute to the pathogenesis of RA. This study has the limitation of a low number of patients studied, and hence the role of γδ T cells should be investigated in a larger cohort of patients, including analysis of γδ T cells at the site of disease. Moreover, additional miRNA silencing experimental approaches are needed to prove the role of these miRNAs in the regulation of Vγ9Vδ2 T cells [26].

## Abbreviations

ACPA: Anti-citrullinated protein antibody
APC: Allophycocyanin
DMARD: Disease-modifying anti-rheumatic drug
FITC: Fluorescein isothiocyanate
HD: Healthy donors
mAbs: Monoclonal antibodies
miRNA: MicroRNA
ncRNA: Non-coding RNA
PBMC: Peripheral blood mononuclear cells
PDCD4: Programmed cell death 4
PE: Phycoerythrin
PerCP: Peridinin chlorophyll protein
PMA: Phorbolmyristate acetate
RA: Rheumatoid arthritis
RT-PCR: Real-time polymerase chain reaction
T_CM_: T central memory
T_EM_: T effector memory
T_EMRA_: T terminally differentiated effector memory
Th: T helper
T_naive_: T naive
Treg: Regulatory T
